# Intensity of Territorial Marking Predicts Wolf Reproduction: Implications for Wolf Monitoring

**DOI:** 10.1371/journal.pone.0093015

**Published:** 2014-03-24

**Authors:** Luis Llaneza, Emilio J. García, José Vicente López-Bao

**Affiliations:** 1 A.RE.NA. Asesores en Recursos Naturales, S.L., Lugo, Spain; 2 Research Unit of Biodiversity (UO/CSIC/PA), Oviedo University, Mieres, Spain; 3 Grimsö Wildlife Research Station, Swedish University of Agricultural Sciences (SLU), Riddarhyttan, Sweden; Institut Pluridisciplinaire Hubert Curien, France

## Abstract

**Background:**

The implementation of intensive and complex approaches to monitor large carnivores is resource demanding, restricted to endangered species, small populations, or small distribution ranges. Wolf monitoring over large spatial scales is difficult, but the management of such contentious species requires regular estimations of abundance to guide decision-makers. The integration of wolf marking behaviour with simple sign counts may offer a cost-effective alternative to monitor the status of wolf populations over large spatial scales.

**Methodology/Principal Findings:**

We used a multi-sampling approach, based on the collection of visual and scent wolf marks (faeces and ground scratching) and the assessment of wolf reproduction using howling and observation points, to test whether the intensity of marking behaviour around the pup-rearing period (summer-autumn) could reflect wolf reproduction. Between 1994 and 2007 we collected 1,964 wolf marks in a total of 1,877 km surveyed and we searched for the pups' presence (1,497 howling and 307 observations points) in 42 sampling sites with a regular presence of wolves (120 sampling sites/year). The number of wolf marks was ca. 3 times higher in sites with a confirmed presence of pups (20.3 vs. 7.2 marks). We found a significant relationship between the number of wolf marks (mean and maximum relative abundance index) and the probability of wolf reproduction.

**Conclusions/Significance:**

This research establishes a real-time relationship between the intensity of wolf marking behaviour and wolf reproduction. We suggest a conservative cutting point of 0.60 for the probability of wolf reproduction to monitor wolves on a regional scale combined with the use of the mean relative abundance index of wolf marks in a given area. We show how the integration of wolf behaviour with simple sampling procedures permit rapid, real-time, and cost-effective assessments of the breeding status of wolf packs with substantial implications to monitor wolves at large spatial scales.

## Introduction

Large carnivores are actively managed in an ebb and flow between human interests and conservation goals. The relative weight of social, economic, conservation or ecological factors [1]–[6] tip the balance towards either side continuously, turning the management of large carnivores into one of the most complex tasks within wildlife management. To achieve an adaptive management approach [7], decision-makers require reliable and updated estimates of their status to establish, for instance, trends, conservation actions, sustainable hunting quotas or population controls [3], [8], [9]. Independent of the target, primary management step is the ability to obtain objective, reliable and economically affordable abundance estimates that are easily reproducible over time.

However, despite the fact that the wide range of methods available to survey these elusive species has [10], [11], significantly increased due to promising advances in non-invasive molecular tools [10], camera trapping [12], and statistical procedures [13], [14], the implementation of complex approaches over large spatial scales (i.e. regions or countries) or large populations of these species is resource demanding. Consequently, these methods are still commonly restricted to threatened species, small populations or small distribution ranges [14]–[16]. Because resources for wildlife management are limited [17] (especially deteriorated under the current global financial crisis) and budgets are generally dependent on the conservation status of species [18], the availability of handsome funds to survey large carnivores over large spatial scales is not always guaranteed. This uncertainty is especially important if species or populations are not threatened or are delisted from any protective conservation status [19]. But, the management of contentious species such as large canids, bears or felids must require regular estimations of their abundance regardless of their conservation status in order to guide decision-makers, especially for those populations living in human-dominated landscapes [20].

As a consequence of multiple conflicts surrounding wolves (*Canis lupus*), a development of simple and efficient tools to regularly assess the status of populations is a pressing need. Methods to survey wolf populations involve sign surveys, howling, radio tracking, questionnaires, or statistics of livestock damages and hunting bags [21]–[23]. More recently, the use of non-invasive samples became popular [24]–[26]. In Europe, wolf management relies on an estimated number of individuals or packs, with the former estimation being less accurate as population size or range increases, and the quality of both wolf estimates varies widely across countries and within populations (see http://www.kora.ch/sp-ois/wolf-ois) [27]. Thus, non-invasive samples or intensive radio-tracking are usually implemented over small spatial scales or small populations (<500 individuals) [16], [24], [26], [28], whereas questionnaires or hunting statistics are commonly used over vast areas and large populations, increasing uncertainty (see http://www.kora.ch/sp-ois/wolf-ois) [27]. On the other hand, small populations are annually surveyed (e.g. Scandinavian or the Western Alps wolf populations) [16], [28], whereas a lack of temporal continuity or partial assessment covering small population fractions (usually corresponding to different administrative limits such as counties or national parks) is normal in large populations. This is the case for the NW Iberian wolf population – the main wolf population in Western Europe [29] - where the last comprehensive view of the status of the whole population (as a result of several Spanish regional surveys and a Portuguese national census) was ten years ago (estimated population size between 1999 and 2003 of ca. 320 packs or ca. 2000–3000 individuals) [30], and only some regional surveys are regularly implemented (e.g. county of Asturias).

Wolf monitoring over large spatial scales is difficult. However, the integration of wolf marking behaviour with simple indexes resulting from sign counts (e.g. visual and scent marks such as faeces) may offer a good alternative to monitor wolves over large areas. Sign counts have been widely used as indexes of relative abundance of large carnivores. Government agencies have implemented them as a relative low-cost approach to gathering data at large scales using rangers, technical staff or volunteers [10], [21]. Although there have been attempts to correlate these indexes with densities [31], few efforts have been carried out in order to link sign counts with demographic parameters such as breeding status. The most popular attempt is the use of snow-tracking to infer reproduction [28], [32]; but it is a time-delayed method dependent on long periods of good snow conditions the next winter after the breeding period, absent, for instance, in most of the southern wolf's European range.

Territoriality in wolves is indicated by the means of visual and scent marks such as faeces, urine, ground scratching or anal gland segregations [32]–[36]. Pack members accumulate these marks, for instance, in the edges of their territories or the surroundings of the *rendezvous sites* [32]–[36]. As a general rule, only mature, dominant wolves, breeds every year within the pack [32], and they show an intense territorial marking behaviour compared to other members of the pack [27], [37]–[39]. Thus, we hypothesised that as pack members, particularly the breeding pair, use visual and scent marks to indicate possession and defence of the pack's territory, high levels of marking behaviour around the breeding season in an area could reflect the existence of an organised pack and a successful breeding pair. Since a biological meaningful units to monitor wolf populations are the pack and the number of wolf reproductions, then high abundance of wolf marks around this period could be used as an indicator of successful reproduction (i.e. the presence of pups) [39] and may offer a cost-effective tool to monitor wolves (i.e. number of wolf reproductions). However, to date, no studies have attempted to establish a real-time link between wolf marking behaviour and breeding status in a pack [39]. We discuss the application of the method proposed here for wolf monitoring over large spatial scales (i.e. countries or entire wolf populations).

## Methods

### Study area

**Figure 1:**
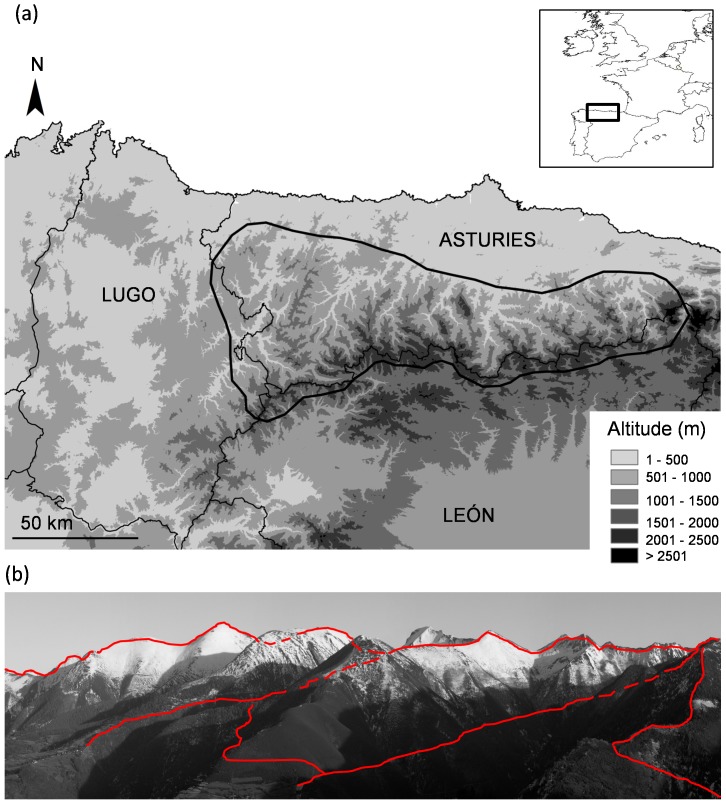
Study area and sampling design. (a) Map showing the study area located in the Cantabrian Mountains (N Spain). (b) Scheme showing the spatial distribution of transects within a typical sampling site.

The study was carried out in the Cantabrian Mountains, N Iberia (ca. 9,000 km^2^; Fig. 1a), covering the rugged region of Asturias and a small mountain area within Galicia (east of Lugo province, hereafter Lugo; Fig. 1a). Vegetation is mainly comprised by scrublands, woodlands, and grasslands (pastures). Scrublands are mainly composed by *Calluna vulgaris*, *Genista* spp., *Erica* spp. and *Vaccinium* spp..Woodlands are dominated by beech (*Fagus sylvatica*), oaks (mainly *Quercus petraea* and *Quercus pyrenaica*), birch (*Betula pubescens*) and anthropogenic chesnut trees (*Castanea sativa*). Isolated trees or small groups of holly (*Ilex aquifolium*) and rowan (*Sorbus aucuparia*) often occur scattered through mature or secondary forests. Scrublands are the predominant habitat type and the level of fragmentation of forests here is high [40]. Snow is present irregularly (both seasonally and annually) between December and March.

### Collection of wolf marks

We used 42 sampling sites with a regular presence of wolves during the last two decades (39 in Asturias and 3 in Lugo). Each sampling site comprised 2–5 valleys and the surrounding mountains covering approximately 150–200 km^2^ (see Fig. 1b). This area was of a similar size than the average home range size reported in wolves in N Iberia [41]. All sites were regularly sampled between 1994 and 2007 around the pup-rearing period (summer-autumn) within different regional wolf surveys (from 1994 to 2004 in Asturias and between 2006 and 2007 in Lugo). Information from these surveys was comparable since the same field procedures (see details below) were implemented in all surveys throughout the study period [42]. Each sampling site was surveyed on average 3 years (range 1–6), with a total of 120 sampling sites/year (hereafter sites, note that this is the sample size that will be used for subsequent analyses). Within each site, wolf marks were searched in transects along existing paths and trails, on foot or using a vehicle (<10 km h^−1^) covering as much selected area as possible (see Fig. 1b).

Faeces and ground scratching marks serve as visual and scent marks in wolves [34]–[36]. Based on their visual function facilitating their detection by field observers with different levels of expertise compared with urine –which is also dependent on snow conditions-, we considered both visual wolf marks for this study (hereafter wolf marks). Shape, size, content, smell and spatial position (i.e. distance to villages) were, in combination, diagnostic attributes to determine wolf faeces. In those cases that there were clear doubts on the identity of the species, faeces were not collected. We considered the criteria used to identify wolf faeces and the experience of field-observers reliable since a posterior trial determining wolf faeces using these diagnostic attributes and a parallel DNA analyses confirmed that 90% of faeces were correctly assigned to wolves (n = 108, *unpublished data*). On the other hand, ground scratching marks were identified on the basis of size, length, intensity (i.e. excluding those marks compatible with dogs) [36]. The presence of wolf faeces accompanying this sign was an important factor determining wolf scratching.

As random sampling is not effective to locate wolf marks [42], surveys in this mountainous region were focused on landscape elements often used by wolves as marking places (paths and trails, particularly focusing on junctions and mountain passes; see Fig. 1b) [34], [42], [43] where the probability of the detection of wolf marks by other intra and inter-pack individuals is maximized. Mountain passes may favour wolf movements between valleys in mountainous areas turning these places into important landmarks on a landscape level. One to several wildlife trails/paths usually cross mountain passes being an important place for territorial marking. For example, considering 10 transects crossing such passes in our study area, out of the 164 wolf marks found in a total of 83.5 km surveyed (an average of 8.5 km per transect), 82% of wolf marks were located in these particular conspicuous sites (ranging from 40% to 100% of wolf marks detected per transect).

The ability of wolves to deposit their marks in conspicuous sites (both on a large and fine spatial scale) [34], [35], [39], [43] indicates a degree of control about where to locate their marks. For example, wolf faeces, whether deposited as scent marks or not, are recognized as important visual marks and a powerful sources of odour [34], [35], [39]. Although it is difficult to distinguish between faeces with and without (i.e. excretion) an intention of communication [35], [38], pack members seems to use substrates and conspicuous places differently according to their position in the social hierarchy [38], and some faeces perhaps had other functions than territorial marking during the period of study (e.g. for marking empty food caches) [35], [39], we considered that all faeces placed on the abovementioned landscape features represented some kind of marking behaviour [39]. This assumption also relies on the fact that wolf marks were deposited on conspicuous places on a landscape level that enhance them as marks [32], [35], [36], [39], [43]. This procedure decrease the probability to include faeces from young and lone wolves since they located more often their faeces off-trail [25], [26], [39]. Potential differences in marking behaviour among pack members [38] were not considered in this study. Furthermore, considering the posterior spatial distribution of rendezvous sites -based on the results from the howling and observation points - transects did not cross rendezvous sites, decreasing the probability to count pup faeces as wolf marks as well as avoiding the inclusion of notable aggregations of faeces in these sites as a consequence of its regular use by pack members (e.g. resting sites) [32].

Taking into account the landscape configuration and the availability of paths and trails, 1 to 9 transects were sampled within each site with an average number of 4 transects per site (median  = 4), varying in length from 1 to 18 km (average length per transect  = 4.1 km, n_transects_ = 463). All transects were conducted once around the pup rearing period. Seventy-seven percent of transects were surveyed between August and September, when all individuals of the pack are relatively stable around rendezvous sites [32]. The remaining surveys were carried out between October and early November. The number of wolf marks by transect was noted.

### Reproductive success

Two complementary methods, simulated howling and direct observation points, were used to assess wolf reproduction (i.e. presence of pups in the rendezvous sites). First, human-simulated howling elicited the response of adults and pups in the rendezvous site [44]. In each howling point, the observer emitted between 3 to 5 howls, each separated between 5 and 8 s long breaks [44]. If wolves did not reply within 2 minutes, then the observer repeated this process 2–3 times. The selection of the howling points was based on the landscape configuration (e.g. distribution of potential rendezvous sites based on the availability of refuge and areas with low human activity) [45], the environmental conditions (selection of the best places ensuring optimal conditions to simulate howling and to hear replies), and information gathered previously in the collection of wolf marks [42]. Howling sessions started at sunset and spanned the early night-time hours, avoiding rainy or windy nights, and were carried out between August and October when reply rate is remarkable [44] (occasionally early November). Second, observation points were used to contact pups in the rendezvous sites. The observer used 8× or 10× binoculars and telescopes with 20–60× zoom lenses to scan potential rendezvous sites and the surrounding areas during at least one hour. Observation points were carried out at sunrise and sunset in the same period of howling sessions.

We only considered reproduction when pups responded positively to howling or when they were observed at the rendezvous site or its surroundings. Sampling effort was nearly constant among sites with four days invested per site; which overall, accounted for 480 days along the study period.

### Ethics statement

In Spain, wolves north of river Duero are in Annex V of the European Habitats Directive (92/43/EEC); being a game species in Galicia and a species with a special regime (no game species) in Asturias. Fieldwork procedures (collection of wolf marks and the methods used to confirm the presence of pups) were specifically approved by the Regional Governments of Asturias and Galicia as well as the Spanish Ministry of Environment (Picos de Europa National Park) as a part of regional and local wolf monitoring activities. We did not handle any individual.

### Data analyses

For each site, we considered two measures of relative abundance of wolf marks. First, we calculated a mean relative index of abundance of wolf marks as the ratio between the total number of marks found and the sum of the length of all surveyed transects (in kilometres). Second, we identified the transect with the highest value of relative index of abundance (i.e. the ratio between the marks found and the length for each transect) and we considered this value as the maximum relative index of abundance per site. Moreover, we categorised all sites in a binary variable, according to the confirmed presence of pups.

Differences in sampling effort among sites could affect either the value of the relative indexes of abundance used or the probability to detect wolf reproduction, which could ultimately affect our results. Therefore, we first tested whether the sampling effort was different between sites with and without confirmed wolf reproduction by comparing transect length as well as the number of howling and observation points using Mann-Whitney U tests. While the former comparison gives us an idea about the homogeneity in the sampling effort to find wolf marks irrespective of the presence of pups, the two other comparisons inform how invested the effort in detecting pups was. We assumed higher values of howling points in areas without confirmed wolf reproduction would reduce the probability of false absences of pups.

We then evaluated the relationship between wolf reproduction (i.e. the presence of pups) and the mean and the maximum relative indexes of abundance of wolf marks (predictors). Since the same site was sampled several years, we built generalised linear mixed models (GLMMs) with binomial error distribution and logit link. We included site and year as random factors in the models. Although sampling effort per site (total transect length) did not differ between sites with and without a confirmed presence of pups or between months, its variance was considerable (72 km), which could potentially affect the studied relationships. Therefore, we included in the models a covariate, the total transect length, in order to statistically control their potential effect. Additionally, we included a second covariate in the models (month) to account for temporal differences in wolf mark detectability and abundance. Finally, as total transect length per site positively correlated with the number of transects (Spearman coefficient correlation, rs = 0.845, *P*<0.0001) and the number of transects per site could influence the maximum relative index of abundance (the probability to find higher indexes increase with the number of transects), we included this factor as another covariate in the model with the maximum relative index of abundance. For each model, we estimated the marginal and the conditional R^2^ following Nakagawa and Schielzeth (2013) [46]. Marginal R^2^ represents the variance explained by fixed factors (relative indexes of abundance of wolf marks, total transect length, month, number of transects per site) whereas Conditional R^2^ is interpreted as variance explained by both fixed and random factors. We predicted the probability of wolf reproduction based on the intensity of wolf marking behaviour and determined the cutting points for both relative abundance indexes when the probability of wolf reproduction was higher than 0.60, 0.80 and 0.99 (these values were selected according to their informative and conservative value from a management point of view). All GLMMs were fitted with SAS 9.2 (procedure GLIMMIX) [47].

## Results

A total of 1,877 km were surveyed in 463 transects with an average length (± s.d.) of transects of 15.6±8.5 km per site. Overall, 1,964 wolf marks were found and 1,497 howling (mean number per site  = 12.5, range 1–43) and 307 observations points (mean number per site  = 2.5, range 1–18) were used to confirm the presence of pups. Out of the 120 sites surveyed, wolf reproduction was confirmed in 84 cases (70%).

Comparing sites with and without reproduction, average total transect length was similar, 14.8 km and 17.7 km respectively, and we did not detect significant differences between groups (Mann-Whitney U test, *P* = 0.151, n = 120), indicating homogeneity in the sampling effort invested among sites in relation with the number of kilometers surveyed. However, the number of howling and observation points was different between groups. Whereas the mean number of howling points was significantly higher in sites without a confirmed reproduction (16 *vs.* 11; Mann-Whitney U test, *P* = 0.005, n = 120); the number of observation points showed the opposite pattern (2 *vs.* 3; Mann-Whitney U test, *P* = 0.029, n = 120). Although observation points were made in all sites regardless of the confirmation of reproduction, they particularly confirmed wolf reproduction in those sites where howling sessions were positive.

**Figure 2:**
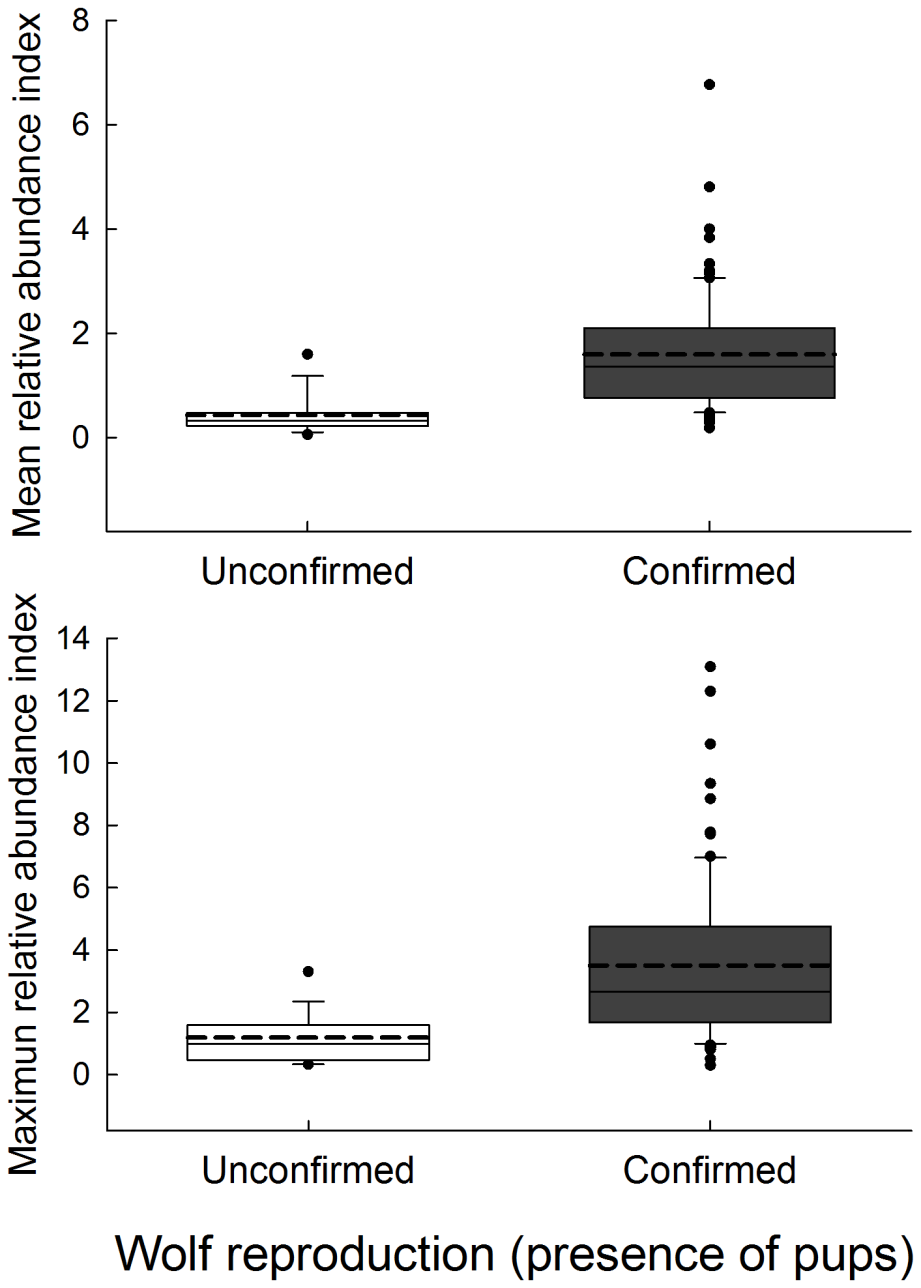
Box-plot showing mean and maximum relative abundance indexes of wolf marks in sites with and without confirmed wolf reproduction (presence of pups). Grey box indicates sites with confirmed wolf reproduction, whereas white box refers to sites without confirmed wolf reproduction. The continuous line within the box indicate the median; whereas the discontinuous line shows the mean value. The lower end of the box indicate the 25^th^ percentile, the upper end of the box indicate the 75^th^ percentile, and the error bars shows the 10^th^ and 90^th^ percentiles. Points denote outliers.

**Figure 3:**
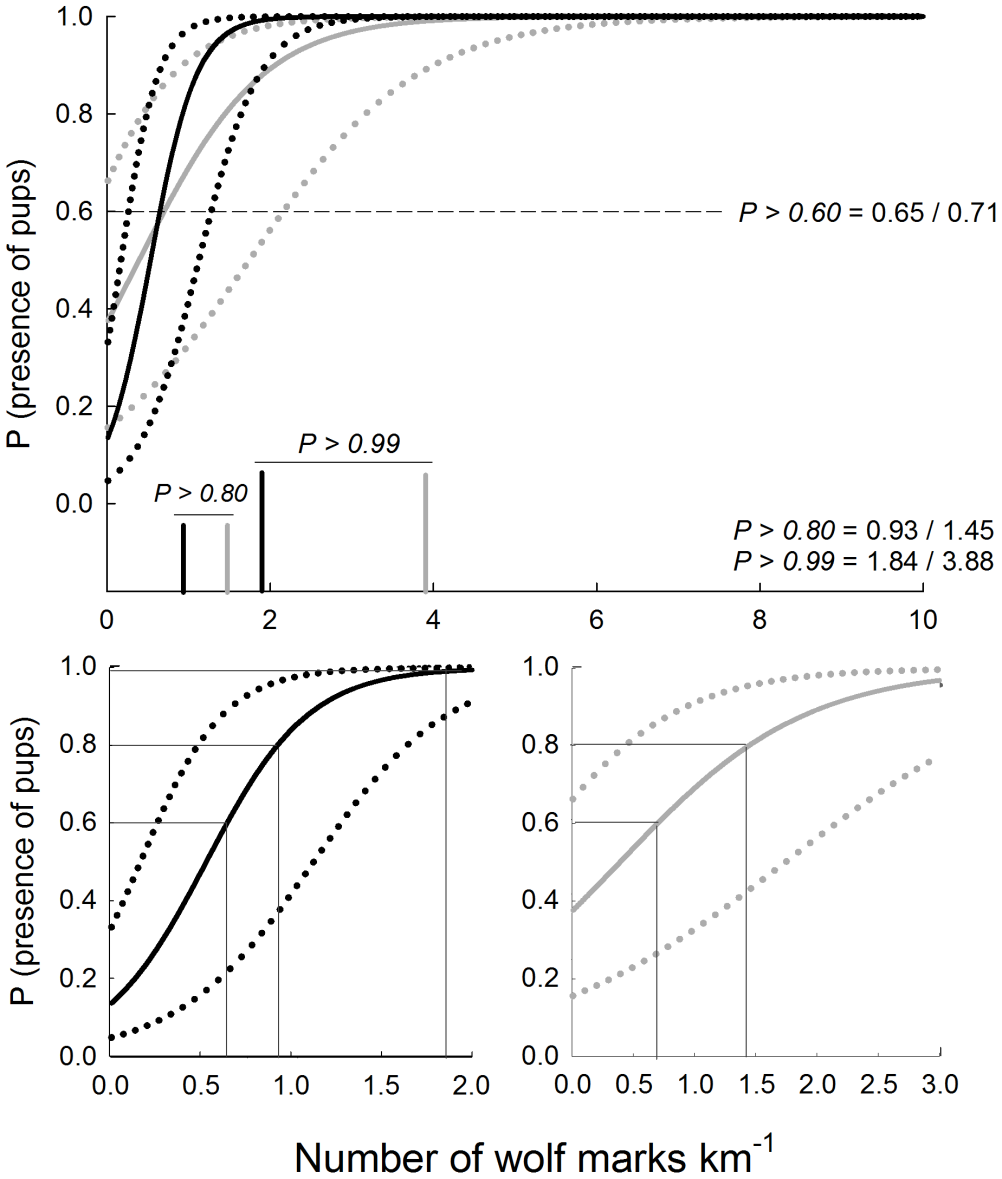
Predicted probability of presence of pups against the mean and the maximum relative abundance index of wolf marks. Continuous black and grey line refers to the mean and the maximum relative abundance index of wolf marks, respectively (± s.e., dotted lines). Striped line show the cutting point of 0.60; whereas the vertical black and grey bars show the cutting points of 0.80 and 0.99 for the mean and the maximum relative abundance index, respectively. Numbers for these cutting points refers to the number of wolf marks km^−1^ for the mean (left) and the maximum (right) relative abundance index. For clarity, we also show a zoom (bottom) on these predicted probabilities, with lines denoting the correspondence between the established cutting points and the values of the relative abundance indexes, for the first range of values of the number of wolf marks km^−1^.

The mean number of wolf marks was ca. 3 times higher in sites with a confirmed presence of pups (20.3 *vs.* 7.2 marks in sites with and without confirmed reproduction, respectively) with the mean relative index of abundance lower in sites without confirmed reproduction (0.43; n = 36) compared to sites with confirmed reproduction (1.61; n = 84; Fig. 2). The same pattern was observed for the maximum relative index of abundance (1.19 *vs.* 3.16 respectively; Fig. 2). We found a significant relationship between the mean and the maximum relative abundance index and the probability of wolf reproduction (χ2*^2^* = 17.52, d.f. = 1, *P<0.0001* and χ2 = 14.55, d.f.  = 1, *P<0.001*, respectively; Table 1; Fig. 3) when month, total transect length and number of transects were controlled for (all covariates with a *P*>0.285; Table 1). The model using the mean relative abundance index of wolf marks showed a marginal R^2^ of 0.79 and a conditional R^2^ of 0.84, whereas in the case of the model considering the maximum relative abundance of wolf marks, marginal R^2^ was 0.63 and conditional R^2^ was 0.77. These results indicate that the explained variance by the random factors (pack and year) was small.

**Table 1:** Parameter estimates (± s.e.) in the models testing the relationship between the mean and the maximum relative abundance of wolf marks (marks km^−1^) and wolf reproduction.

		Mean relative abundance index	Maximum relative abundance index
Model-effect		Parameter estimate (± s.e.)	Parameter estimate (± s.e.)
Intercept		−1.88±1.14	−0.51±1.17
Relative abundance of wolf marks		3.53±0.84*	1.32±0.34*
Sampling effort (km)		−0.01±0.04	0.05±0.07
Number of transects		-	−0.21±0.30
Month	*September*	0.51±0.84	0.99±0.87
	*October*	0.39±1.07	0.79±1.05
	*November*	−1.48±1.27	−0.77±1.21

The level “month (August)” is included in the intercept.

* Significant at P<0.001.

*Note that the covariate “number of transects” was not included in the model with the mean relative abundance index (see text for details).*

Our models predicted a probability of wolf reproduction higher than 0.60, 0.80 and 0.99 for a mean relative abundance index of 0.65, 0.93 and 1.84 wolf marks per km respectively; and a maximum relative abundance index of 0.71, 1.45 and 3.88 marks per km, respectively (Fig. 3). Predicted probabilities of pup presence by both indexes were positively correlated (Spearman coefficient correlation, rs  = 0.843, *P*<0.0001). In fact, considering the three established cutting points and focusing on the mean relative abundance index, we observed 100% of agreement with the maximum relative abundance index in determining wolf reproduction for a probability of 0.60, 97% for a probability of 0.80, and 96% for a probability of 0.99. The proportion of false positives increased towards lower values of cutting points for the probability of pup presence (Table 2). Within each selected cutting point, this proportion was always higher for the maximum relative abundance index (Table 2), although differences were not significant (Z-test, all *P-values*>0.100; Table 2).

**Table 2:** Proportion of false positives (Type I error) using the 0.60, 0.80 and 0.99 cutting points for the predicted probability of wolf reproduction based on the mean and the maximum relative abundance index.

	Mean relative abundance index	Maximum relative abundance index
Cutting point	Proportion of false positives (number of total cases)	Proportion of false positives (number of total cases)
0.60	0.08 (75)	0.18 (101)
0.80	0.07 (61)	0.14 (80)
0.99	0.00 (26)	0.04 (22)

## Discussion

### Wolf marking behaviour and sign counts

Individual asymmetries in territorial marking behaviour can influence indices of abundance based on marks [25], [26], [48], [49]. Thus, understanding the link between different marking behaviours and the abundance of marks is the only way to efficiently interpret monitoring procedures based on marks (i.e. sign counts). Territoriality in carnivorous mammals is commonly indicated by using scent or visual marking, such as urine, faeces or ground scratching, playing these marks an important role in territory maintenance and defence, indication of breeding status, demarcation of valuable places such as rich-food patches or home sites and intra- or inter-specific communication [34]–[36], [50]. Reproductively active individuals - dominants in social species - assume a high cost in territorial marking behaviour compared to subordinates [38], [51].

In the case of wolves, an assumption that only the mature pair breeds [32], translates into a higher territorial marking intensity by the breeding individuals [26], [38], [45], especially in conspicuous sites, to increase the probability of the mark's detection by conspecifics. On the contrary, lone wolves or young individuals tend to deposit less marks in these conspicuous sites compared to breeding wolves and other pack members [25], [32], [39]. In the case of lone wolves this behaviour may be particular important to decrease the probability of an encounter with territory holders. Higher levels of sex hormones detected in the wolf faeces located in particular conspicuous sites such as junctions supports these behavioural patterns [38]. As predicted, high levels of wolf marking behaviour around breeding season (pup rearing period) will reflect the existence of a successful breeding pair and the presence of pups. The observation that the number of wolf marks was three times higher in sites with a confirmed presence of pups compared with sites where wolves were present but pups were not confirmed is worth mentioning. Because pups are mainly stationary around rendezvous sites in the first months of life, they can not be responsible for a higher number of faeces in these areas. On the other hand, an absence of temporal differences in the intensity of territorial marking (we only detected a non-significant decrease in November, see Table 1) suggests constancy in this behaviour during this period.

### Determining wolf reproduction

Simple mark/sign counts provide valuable information about presence, distribution or relative abundance of large carnivores [10]. However, gathering data on demographic parameters generally required other intensive, invasive, and expensive approaches (e.g. radiotracking or DNA analyses), often feasible for research-oriented efforts applied over small areas or small populations. Excluding snow-tracking – a time-delayed approach used to determine the occurrence of reproduction events based on the number of individuals detected (tracks) in the following winter - which shows several logistical constraints associated to snow conditions, spatial scale (high cost and significant field effort needed) and, sometimes, interpretation [28], to our knowledge, this is the first time that it is established a real-time relationship between intensity of territorial marking and the probability of wolf reproduction.

The level of confidence determining the probability of wolf reproduction in a given area could be adapted according to different management goals and population scenarios. For example, we recommend a reliable level in small populations (cutting points of 0.80 or 0.99) decreasing the probability of false positives (type I error, see Table 2), whereas this probability could be conservative (0.60) at large populations. The mean relative abundance index showed a low probability of false positive (below 0.10 for all cutting points); being zero for the cutting point of 0.99. Thus, as a general rule, we suggest a cutting point of 0.60 in the probability of wolf reproduction to monitor wolves on a regional scale and the use of the mean instead of the maximum relative abundance index of wolf marks. This option will reduce the influence of short transects with a high number of wolf marks or a small number of transects per site on the probability of wolf reproduction. By combining this method with different spatial criteria, such as the distance among packs in different scenarios (saturated, non-saturated or recovering areas) or simulated wolf territories, it would be possible to improve the estimates on the number of wolf packs. In addition, we highlight that a similar approach based on wolf marking behaviour could determine the probability of the occurrence of non-breeding packs. This application would require the understanding of differences in the intensity of territorial marking in areas with lonely or floaters individuals and areas with established non-breeding pairs or family groups.

Although our analyses were controlled for mark detectability, we acknowledge that prior to generalising the use of this method, it is necessary to develop similar trials in other landscape configurations and wolf populations evaluating quantitative changes in the values of the relative abundance indexes in established cutting points for the probability of wolf reproduction (0.60, 0.80 and 0.99). For example, several studies suggest that wolves use linear infrastructures to place a significant proportion of their territorial marks [31], [32], [34], [42], [43], but how landscape configuration influences this marking behaviour is unknown. Another important concern in the use of this method would be the feasibility in the identification of wolf marks. However, features described above may be efficiently used as diagnostic attributes, along with the training of observers, resulting in high correct assignment rates [52].

On the other hand, even although we used a multiple sampling approach to determine reproduction events, it is well-known that both methods are not completely efficient [53], [54], and the existence of an important number of false absences could affect our results. However, in our case, sampling effort was higher in those sites where pups were not detected, decreasing the probability of false absences of wolf reproduction. In fact, for both relative abundance indexes, the 25^th^ percentiles of sites with confirmed wolf reproduction did not overlap or showed a low overlapping with the 75^th^ and 90^th^ percentiles, respectively, of sites with unconfirmed wolf reproduction (Fig. 2), suggesting that the number of false absences was small. Finally, since the probability of wolf reproduction was independent of a sampling effort, indicating the importance of a stratified sampling to monitor wolves based on wolf marking behaviour, more studies are needed to optimize sampling design (e.g. minimum transect length per site). Moreover, because pack members seems to use substrates and conspicuous places differently for marking behaviour [38], the influence of different levels of territorial marking, according to individual attributes and the conspicuous sites considered, on the studied relationships deserves further investigation.

Our findings would objectively reduce the level of uncertainty in the estimation of the number of packs, particularly when they are not confirmed with other procedures [53]. For example, many monitoring programmes simulate howls to locate pups [22], [23], but this method also has drawbacks in relation with the subjectivity of the observer determining the participation of pups in a chorus howl and the fact that wolves occasionally do not respond, as well as its logistical constraints when it is applied over large scales [54]. For wolf surveys covering large areas, it has been suggested that the effectiveness of simulated howling could be improved by selecting howling sites in those places with the largest concentrations of wolf marks during the breeding period [42]. In other words, selecting sites where the probability of wolf reproduction is high based on the intensity of territorial marking as this study shows. In wolf surveys based on howling, this method could add an objective approach to determine the probability of wolf reproduction when high relative abundance of wolf marks are found in a given area, but the presence of pups is not verified using howling.

### Surveying wolves at large spatial scales

In the last years, the convenience of estimating the number of wolves or packs (or the number of reproductions) has been the focus of an intense and controversial debate. Finally, wolf biologists and managers seem to agree about the robust and biological meaning of packs as a unit to monitor wolves [16], which could be easily estimated with the method proposed here. However, monitoring and management are interactive processes, and therefore, management goals for each wolf population will determine the level of accuracy required in wolf estimates (individuals or packs). For example, at small populations such as the critically endangered population located in Sierra Morena (S Spain; <50 individuals) [27], [29] the appropriate level of information would estimate both the number of individuals, using intensive approaches and the number of packs. But, the former level of accuracy may be unnecessary at large populations, such as the NW Iberian wolf population, assuming higher levels of uncertainty until the population would not be below a critical value, such as a given annual number of packs, number of reproductions or effective population size (minimum number of breeding pairs). In this regard, adopting a cost effective approach, the number of packs could serve as sentinel information to shift between intensive (individuals and packs) and simple and extensive (packs) monitoring procedures and vice versa.

We propose that this procedure could be easily exported to other wolf populations and countries. Across Europe, transboundary cooperation in wolf management is a pressing need [29]. This fact requires coordination among all authorities and actions including compatibility and/or convergence in monitoring methods and target information. Since this method does not rely upon complex procedures or equipment, it can be relatively straightforward and quick to conduct by rangers, technical staff or volunteers, and easily implemented in routine samplings. The use of the number of packs may turn estimates more homogeneous in a transboundary context.

### Concluding Remarks

We show how the integration of wolf marking behaviour with simple sampling procedures (sign counts) permit rapid, real-time, and cost effective assessments of the breeding status of wolf packs with substantial implications to monitor wolves over large spatial scales. The assessment of the number of packs using this approach would provide a simple way to regularly assess the status of wolf populations providing acceptable estimates of demographic parameters such as reproduction success or annual effective population sizes. This information is essential for an efficient, adaptive management framework in wolf populations, even when populations are not threatened.

## References

[1] Johansson M, Karlsson J, Pedersen E, Flykt A (2012) Factors Governing Human Fear of Brown Bear and Wolf. *Hum Dimens Wildl* 17: 58–74.

[2] Thirgood S, Woodroffe R, Rabinowitz A (2005) The impact of human-wildlife conflict on human lives and livelihoods. In: Woodroffe R, Thirgood S, Rabinowitz A, editors. People and Wildlife, Conflict or Coexistence? Cambridge University Press, Cambridge, UK. pp. 13–26.

[3] Treves A (2009) Hunting for large carnivore conservation. *J Appl Ecol* 46: 1350–1356.

[4] Mech LD (1995) The challenge and opportunity of recovering wolf populations. *Conserv Biol* 9: 270–278.

[5] Mech LD (2012) Is science in danger of sanctifying the wolf? *Biol Cons* 150: 143–149.

[6] López-Bao JV, Sazatornil V, Llaneza L, Rodríguez A (2013) Indirect effects on heathland conservation and wolf persistence of contradictory policies that threaten traditional free-ranging horse husbandry. *Conserv Lett* 6: 448–455.

[7] Holling CS (1978) Adaptive environmental assessment and management. John Wiley & Sons, New York.

[8] Jhala Y, Qureshi Q, Gopal R (2011) Can the abundance of tigers be assessed from their signs? *J Appl Ecol* 48: 14–24.

[9] Peek J, Dale B, Hristienko H, Kantar L, Loyd KA, et al.. (2012) Management of large mammalian carnivores in North America. The Wildlife Society Technical Review 12–1. The Wildlife Society, Bethesda, Maryland, USA.

[10] Long RA, MacKay P, Zielinski WJ, Ray JC (2008) Noninvasive survey methods for carnivores. Island Press, Washington D.C.

[11] Boitani L, Powell RA (2012) Carnivore ecology and conservation: a handbook of techniques. Oxford University Press, London, UK.

[12] O'Connell AF, Nichols JD, Karanth KU (2011) Camera traps in animal ecology. Methods and analyses. Springer, Tokyo.

[13] Royle JA, Nichols JD, Karanth KU, Gopalaswamy AM (2009) A hierarchical model for estimating density in camera-trap studies. *J Appl Ecol* 46: 118–127.

[14] Karanth KU, Gopalaswamy AM, Kumar NS, Vaidyanathan S, Nichols JD, et al (2011) Monitoring carnivore populations at the landscape scale: occupancy modelling of tigers from sign surveys. *J Appl Ecol* 48: 1048–1056.

[15] Kendall KC, States U, Survey G, Rocky N, Science M, et al (2009) Demography and genetic structure of a recovering grizzly bear population. *J Wildl Manage* 73: 3–17.

[16] Marucco F, Boitani L (2012) Wolf population monitoring and livestock depredation preventive methods in Europe. *Hystrix* 23: 1–4.

[17] Leader-Williams N, Albon SD (1988) Allocation of resources for conservation. *Nature* 336: 533–535.

[18] Palomares F, Rodríguez A, Revilla E, López-Bao JV, Calzada J (2011) Assessment of the conservation efforts to prevent extinction of the Iberian lynx. *Conserv Biol* 25: 4–8. 21091768 10.1111/j.1523-1739.2010.01607.x

[19] Gude JA, Mitchell MS, Russell RE, Sime CE, Bangs EE, et al (2012) Wolf population dynamics in the U.S. Northern Rocky Mountains are affected by recruitment and human-caused mortality. *J Wildl Manage* 76: 108–118.

[20] Llaneza L, López-Bao JV, Sazatornil V (2012) Insights into wolf presence in human-dominated landscapes: the relative role of food availability, humans and landscape attributes. *Divers Distrib* 18: 459–469.

[21] Linnell JDC, Swenson JE, Landa A, Kvam T (1998) Methods for monitoring European large carnivores – A worldwide review of relevant experience. *NINA Oppdragsmelding* 549: 1–38.

[22] Blanco JC, Cortés Y (2002) Ecología, censos, percepción y evolución del lobo en España: análisis de un conflicto. SECEM, Málaga. 176 pp.

[23] Kunkel K, Mack CM, Melquist WE (2005) An Assessment of Current Methods for Surveying and Monitoring Wolves. Report: 1–79. 2005. Lapwai, Idaho, Nez Perce Tribe, Lapwai.

[24] Caniglia R, Fabbri E, Cubaynes S, Randi E (2012) An improved procedure to estimate wolf abundance using non-invasive genetic sampling and capture – recapture mixture models. *Conserv Genet* 13: 53–64.

[25] Marucco F, Pletscher DH, Boitani L, Schwartz MK, Pilgrim KL, et al (2009) Wolf survival and population trend using non-invasive capture–recapture techniques in the Western Alps. *J Appl Ecol* 46: 1003–1010.

[26] Galaverni M, Palumbo D, Fabbri E, Caniglia R, Greco C, et al (2011) Monitoring wolves (*Canis lupus*) by non-invasive genetics and camera trapping: a small-scale pilot study. *Eur J Wildlife Res* 58: 47–58.

[27] Kaczensky P, Chapron G, von Arx M, Huber D, Andrén H, et al.. (2013) Status, management and distribution of large carnivores - bear, lynx, wolf and wolverine - in Europe. Report to the EU Commission, 272 p.

[28] Liberg O, Aronson Å, Sand H, Wabakken P, Maartmann E, et al (2012) Monitoring of wolves in Scandinavia. *Hystrix* 23: 29–34.

[29] Linnell JDC, Boitani L (2012) Building biological realism into wolf management policy: the development of the population approach in Europe. *Hystrix* 23: 80–91.

[30] Alvares F, Barroso I, Blanco JC, Correia J, Cortes Y, et al.. (2005) Wolf status and conservation in theIberianPeninsula. Frontiers of wolf recovery: Southwestern U.S. and the world. Colorado Springs, Colorado.

[31] Crête M, Messier F (1986) Evaluation of indices of gray wolf (*Canis lupus*), density in hardwood-conifer forests of Southwestern Quebec. *Can Field Nat* 101: 147–152.

[32] Mech LD, Boitani L (2003) Wolves: behavior, ecology, conservation. University of Chicago Press.

[33] Kleiman DG (1966) Scent marking in the Canidae. *Symp Zool Soc Lond* 18: 167–177.

[34] Barja I, Miguel FJD, Bárcena F (2005) Faecal marking behaviour of Iberian wolf in different zones of their territory. *Folia Zool* 54: 21–29.

[35] Peters RP, Mech LD (1975) Scent-marking in Wolves. *Am Sci* 63: 628–637. 1200478

[36] Zub K, Theuerkauf J, Jedrzejewski W, Jedrzejewska B, Schmidt K, et al (2003) Wolf pack territory marking in the Bialowieza Primeval Forest (Poland). *Behaviour* 140: 635–648.

[37] Scandura M, Iacolina L, Capitani C, Gazzola A, Mattioli L, et al (2011) Fine-scale genetic structure suggests low levels of short-range gene flow in a wolf population of the Italian Apennines. *Eur J Wildlife Res* 57: 949–958.

[38] Barja I, Silván G, Rosellini S, Piñeiro A, Illera MJ, et al (2008) Quantification of sexual steroid hormones in faeces of Iberian wolf (*Canis lupus signatus*): a non-invasive sex typing method. *Repro Domest Anim* 43: 701–707. 10.1111/j.1439-0531.2007.00974.x 18422862

[39] Rothman RJ, Mech LD (1979) Scent-marking in lone wolves and newly formed pairs. *Anim Behav* 27: 750–760.

[40] García D, Quevedo M, Obeso JR, Abajo A (2005) Fragmentation patterns and protection of montane forest in the Cantabrian range (NW Spain). *Forest Ecol Manag* 208: 29–43.

[41] García E, Llaneza L, Palacios V, López-Bao JV, Sazatornil V, et al.. (2012) Primeros datos sobre la ecología espacial del lobo en Galicia. Abstract-book of the III Iberian Wolf Congress, pp. 44.

[42] Llaneza L, Ordiz A, Palacios V, Uzal A (2005) Monitoring wolf populations using howling points combined with sign survey transects. *W B P* 1: 108–117.

[43] Vilà C, Urios V, Castroviejo J (1994) Use of faeces for scent marking in Iberian wolves (*Canis lupus*). *Can J Zool* 72: 374–377.

[44] Harrington FH, Mech LD (1982) An analysis of howling response parameters useful for wolf pack censusing. *J Wildl Manage* 46: 686–693.

[45] Ausband DE, Mitchell MS, Doherty K, Zager P, Mack CM, et al (2010) Surveying predicted rendezvous sites to monitor gray wolf populations. *J Wildl Manage* 74: 1043–1049.

[46] Nakagawa S, Schielzeth H (2013) A general and simple method for obtaining R*^2^* from Generalized Linear Mixed-effects Models. *Methods Ecol Evol* 4: 133–142.

[47] Littell RC, Milliken GA, Stroup WW, Wolfinger RD, Schabenberger O (2006) SAS for Mixed Models. 2nd ed. SAS Institute Inc., Cary, NC.

[48] Palomares F, Roques S, Chávez C, Silveira L, Keller C, et al (2012) High proportion of male faeces in Jaguar populations. *PLOS ONE* 7: e52923. 23285226 10.1371/journal.pone.0052923 PMC3532461

[49] Ralls K, Sharma S, Smith DA, Bremner-Harrison S, Cypher BL, et al (2010) Changes in Kit Fox Defecation Patterns During the Reproductive Season: Implications for Noninvasive Surveys. *J Wildl Manage* 74: 1457–1462.

[50] Gorman ML (1990) Scent-marking strategies in mammals. *Rev Suisse Zool* 97: 3–29.

[51] Sands J, Creel S (2004) Social dominance, aggression and faecal glucocorticoid levels in a wild population of wolves (*Canis lupus*). *Anim Behav* 67: 387–396.

[52] Godinho R, Castro D, López-Bao JV, García E, Rio-Maior H, et al. (2011) Genetic assessment of error rates in identifying wolf (*Canis lupus*) faeces in four areas of the Iberian Peninsula. Abstract-book of the X Jornadas Españolas de Conservación y Estudio de Mamíferos: p. 64. Fuengirola, Spain.

[53] Blanco JC, Cortés Y (2012) Surveying wolves without snow: a critical review of the methods used in Spain. *Hystrix* 23: 35–48.

[54] Fuller TK, Sarnpson BA (1988) Evaluation of a simulated howling survey for wolves. *J Wildl Manage* 52: 60–63.

